# Scales of Normality: Displays of Extreme Weight and Weight Loss in Blackpool 1920–1940

**DOI:** 10.1080/14780038.2017.1375720

**Published:** 2017-11-03

**Authors:** Emma Purce

**Affiliations:** ^a^ School of History, Rutherford College, University of Kent, Canterbury, UK

**Keywords:** Freak show, seaside, holiday, weight, entertainment

## Abstract

Developments in scientific and medical understanding of disability, as well as an influx of war-disabled veterans in 1914, led to transforming societal attitudes towards those with physical deformities. Thus, scholars suggest that the beginning of the twentieth century witnessed the sudden decline of freak shows as a popular form of entertainment. Whilst freak shows undeniably became less popular in metropolitan spaces such as London or Manchester, they thrived in seaside resorts; sites dedicated to the pursuit of leisure, pleasure and entertainment. This paper examines exhibitions of freakery at British seaside resorts from 1920 to 1940. Through examining the visual material and written reports associated with the displays of unusual bodies at the margins of British life, it argues that seaside freak shows remained centrally important forms of amusement in the twentieth century. Furthermore, it demonstrates how freak shows were constructed utilising public discourse relating to contemporary concepts of health, wellness and the ‘ideal’ or ‘average’ body. Focusing on Blackpool, the foremost health and pleasure resort of the period, this paper analyses exhibitions of extreme weight, to locate their role in the popular construction of ‘normal’ and ‘abnormal’ bodily size within contemporary British culture.

## Introduction

Walking along Blackpool’s ‘Golden Mile’ during the summer seasons of the 1920s and 1930s, holidaymakers were confronted with a variety of stalls and amusements for their entertainment. ‘Starving brides and glass coffins, men with no legs or arms, men with “cork-screw” bodies and three legs, skeletons and pigmies, “tombs of death,” and “dusky dancing beauties”’, provided entertainment for the tourists promenading on Central Beach.^1^ An article in the *Biggleswade Chronicle* revealed the centrality of freak shows to Blackpool’s entertainment environment. It noted,

Outside the Tower, there are amusements galore, all of the customary kind, but there is one group which must be peculiar to Blackpool. Across the top of the first show place were the words, ‘The Lovely Bride lies here starving.’^2^

Popular with many holidaymakers and excursionists, signs boasting of ‘Starving Brides – Sinking FAST!’ and the ‘Fattest Boy Ever Seen’ besieged the front, presenting holidaymakers with the opportunity to appease their curiosity and stare at someone with an unusual body. Displays of extreme weight were both common and popular attractions at seaside resorts. Starvation exhibits were often situated alongside larger performers, such as Lenny Mason ‘The Leicester Fat Boy’ and Miss Rosie, the 36 stone Fat Lady[Fig F0001] [Fig F0002].^3^ Therefore, this establishes that British holidaymakers were fascinated with abnormally sized bodies and that they enjoyed viewing bodily exhibitions as part of their summertime entertainment.

**Figure 1. F0001:**
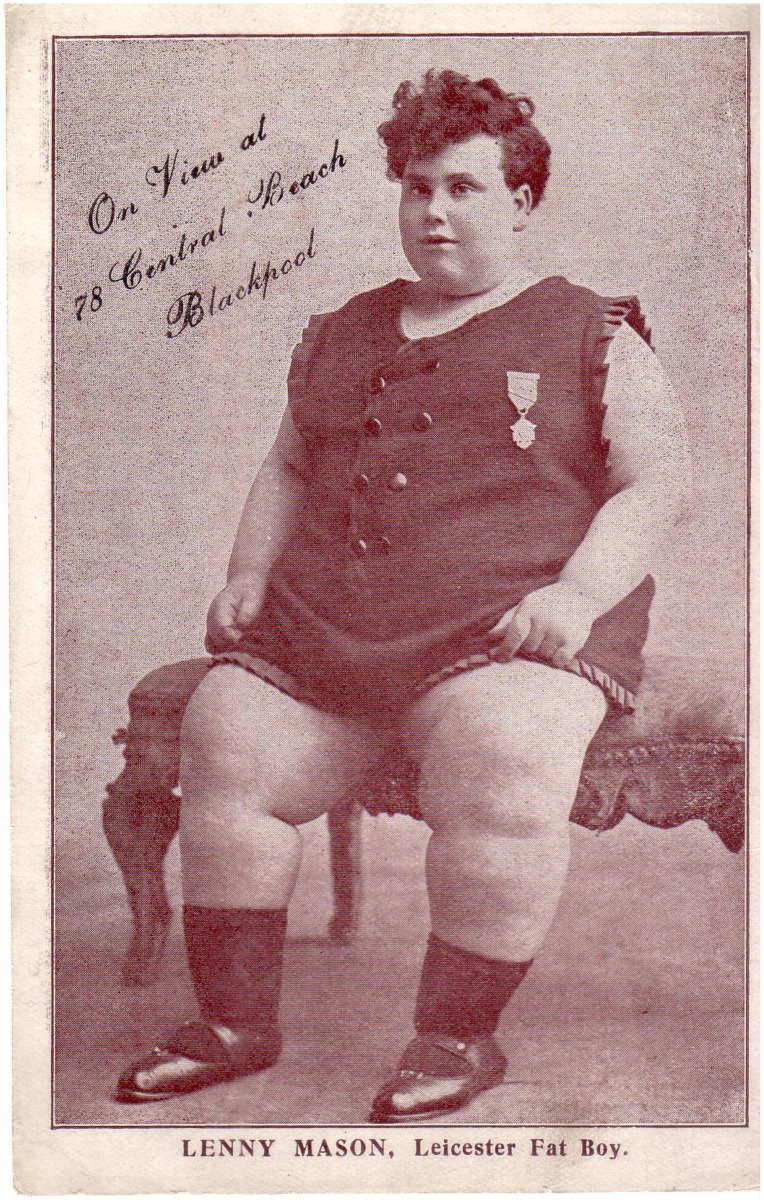
*Carte de Visit* for Lenny Mason, Leicester Fat Boy, Blackpool, The Local and Family History Centre, Blackpool Central Library, Cyril Critchlow Collection, Vol. 163.

Copyright: Endeavoring to find copyright holder for this image.

**Figure 2. F0002:**
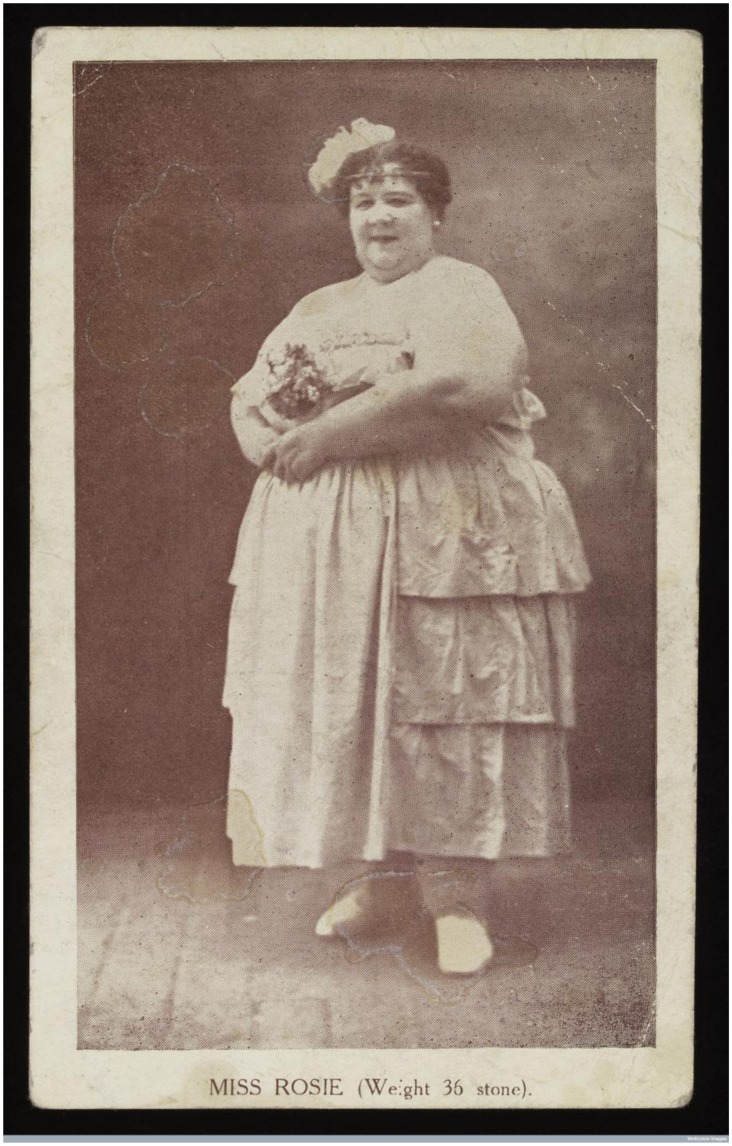
*Carte de Visit* for Miss Rosie, c. 1900, Wellcome Library, London. EPH WD. Freaks:1:1.

Copyright: Wellcome Trust.

This article compares the exhibition of corpulent individuals and starvation performers, exploring the ways in which displays of extreme weight reflected broader conceptualisations of the ‘normal’, ‘average’ or ‘healthy’ body in the first half of the twentieth century. Displays of bodily difference were a cultural institution of the Victorian period.^4^ Subsequently, the wealth of existing scholarship has focused on examining freakery throughout what has been termed the ‘heyday’ of the freak show, from approximately 1847 to 1914.^5^ Nadja Durbach’s seminal text on freak shows in Britain argues that ‘… displays of freakery were critical sites for popular and professional debates about the meanings attached to bodily difference’.^6^ Durbach interprets the freak show as a space in which the British public could make sense of themselves and those around them; as locus of discourse surrounding the perception of ‘normal’, ‘abnormal’, ‘difference’ and ‘deviance’. Examining exhibitions of freakery not only reveals anxieties surrounding the ‘other’ in British society, but additionally, it exposes the ways that the public attempted to normalise themselves in comparison to those on display.

Comparably, Robert Bogdan advocates a social constructionist approach to the history of freakery, and demonstrates that analysing freak shows uncovers attitudes to, and representations of, difference in society and culture. He argues that, ‘“Freak” is not a quality that belongs to the person on display. It is something that we created: a perspective, a set of practices – a social construction’.^7^ Considering the creation of freak shows, through an investigation of the visual depictions and social commentary surrounding the displays, reveals the meanings, representations and constructions of the display for nineteenth century audience members.

At the beginning of the twentieth century, freak shows became less popular in metropolitan areas due to the changing tastes and sensibilities of the public.^8^ However, in seaside resorts, unique cultural environments with their own regulations and customs regarding collective social behaviours, freakery displays continued to be popular amusements. John K. Walton describes the atmosphere and appeal of seaside resorts, ‘as a gateway between land and sea, culture and nature, civilized constraint and liberated hedonism’.^9^ Freed from the cultural limitations of everyday life, the British seaside holiday gave holidaymakers the freedom to have fun and participate in activities that were considered salacious in urban spaces. Although the focus of coastal locations transitioned from health to entertainment, perceptions of health and wellness remained associated with certain activities and amusements available at these places.^10^ It was within this environment that those of extreme weight proved fascinating to the tourists who frequented such exhibitions.

Blackpool was the most popular seaside location in Britain, attracting the upper and working classes alike. Subsequently, it became one of Britain’s best known entertainment institutions. John K. Walton notes that it was ‘Britain’s largest, brashest, busiest and best-publicised popular resort’.^11^ The Golden Mile and Pleasure Beach were iconic sites of the holidaying public and there was comment on the resort at a national level, demonstrating its centrality to British leisure culture.^12^ Locations such as Blackpool were contested spaces of health and entertainment; freak shows encapsulated elements of both. The enmeshing of health and entertainment, within the marginal seaside environment, encouraged the continuation of freak shows in such spaces. Through examining the perpetuation of freak shows in British culture, such as those at seaside resorts akin to Blackpool, we can better understand the centrality of freakery as facilitating public perceptions and representations of difference, deviance and abnormality.

Utilising Blackpool as a case study, due to its importance in British leisure and entertainment history, this article argues that exhibitions of extreme weight provided holidaymakers with a metaphorical ‘scale of normality’ that placed the excessively thin and the grossly obese at either end. It asserts that audiences examined their own bodies and compared them with those on display, thereby discovering whether their personal physique could be considered ‘average’. Consequently, the article suggests that the cultural representations of fatness and thinness in the seaside freak show were situated within broader discourses of health, weight, wellness and ‘normality’, and that this had meaning and significance for the holidaymakers who viewed the exhibitions. Firstly, the article tracks the continuities and changes in contemporary health advice throughout the twentieth century. Secondly, it reveals how health advice changed the cultural construction of ‘normal’ and ‘abnormal’ bodies. Finally, it explores the interrelationship between space, health discourse and the cultural construction of unusual bodies throughout the 1920s and 1930s, including how this was translated to members of the public.

## Health in the Twentieth Century

Throughout the twentieth century, the health of the public caused considerable national anxiety. The 1899 Boer War revealed the poor physical condition of large numbers of the male population and sparked concerns surrounding the degeneration of British society. The higher rate of unfit recruits from large urban towns and cities solidified the belief that industrial life had a detrimental effect on the health of the nation. There was increasing awareness of the need to maintain a healthy body, and by extension, a healthy population. Joanna Bourke suggests that this had a militaristic subtext, whereby the public needed to maintain peak physical condition, due to the substantial threat of war.^13^ There were a number of schemes that endorsed physical fitness, healthy nutrition and exposure to the natural elements as antidotes to ill health. Two main contributors shaped health and health policy in the early decades of the twentieth century; the state, which responded to the revelations of poor health following the Boer War and a number of voluntary organisations, which were committed to improving health through a variety of fitness schemes.

In 1903, the Committee on Physical Deterioration was established in an attempt to improve the health of the nation. While it found little consequential evidence to explain the supposed deterioration, it did encourage a number of initiatives to improve public health, such as medical checks and free school meals for poorer children.^14^

The health of workers was of prime importance to the state, reflected by the passing of the National Insurance Act in 1911. The act promoted social welfare benefits and permitted workers to take paid sick leave. The health of people in the workplace was regarded as integral to preserving the health of the nation, and was the focus of state involvement following the experience of the Boer War.^15^ A preventative approach to illness was advocated in working environments; with nutrition, supplemented by exercise (such as walking to work), encouraged as a way to improve health, increase appetite, and save money.^16^

A more centralised state programme was implemented through the creation of the Ministry of Health in 1919. The Ministry’s first Chief Medical Officer, George Newman, came to office with a clear holistic vision of the path the new ministry should follow. He endorsed a combination of traditional environmental measures and called for a new emphasis on personal health and healthy living.^17^ However, state involvement in improving national health was restricted by financial concerns (particularly following the First World War), and therefore, supplementation from voluntary organisations was influential in the fortification of healthy lifestyles.

Health was so prominent in public discourse that even holidays provided people with healthy activities to enhance their overall well-being.^18^ British seaside resorts, the most popular leisure spaces of all, promoted health through encouraging people to visit the coast on their summer holidays. While modern urban environments were concomitant with unhealthiness, seaside resorts continued to be considered ‘healthy’ spaces. Health recommendations were comprised of a number of principles, including the beneficence of exposure to sunlight, air and water, and the importance of maintaining a healthy body weight.^19^ Coastal locations were influential in promoting knowledge surrounding wellness to the public and many advertisements that recommended seaside holidays endorsed the healthful qualities of the resorts. Seaside holidays were regarded as advantageous to the health of the nation, as they gave people the space to enjoy the healthful benefits of the outdoors. Predominantly, visitors were encouraged to improve their well-being by exposing themselves to the restorative properties of the natural environment. Not only could they participate in swimming and other active pursuits, but they could also benefit from a seaside diet of fish and chips, which was deemed healthy for working-class people. As Walton suggests, the widespread assertion that working-class people were malnourished was rectified in the seaside environment, as fish and chips provided them with a cheap and nutritious meal.^20^ This replicated the idea that both fitness and diet were equally important for comprehensive healthiness.

Ultimately, the improvement and maintenance of bodily health became ever more important in a society concerned by the physical realities exposed during the Boer War. Within this wider debate, the British seaside resort appeared as an integral site to the discussion, promotion and enacting of concepts of well-being. Thus, in the coastal space, the public was encouraged to understand their health better.

## The Cultural Construction of ‘Normal’ and ‘Abnormal’ Bodies

With a greater emphasis placed on wellness, it became more important to procure a statistically ‘normal’ body, as measurements became prominent markers of physical and social health.^21^ From as early as the 1850s, the term ‘normal’ infiltrated the English language. However, it was not laden with the didactic nuances that became synonymous with such terminology until the start of the twentieth century.^22^ Rather, ‘normal’ or ‘abnormal’ were solely expressions associated with statistical measurements of the body. Waltraud Ernst contends,

… although no moral prescription appears to be inherent in this seemingly purely mathematical definition, the seed of evaluation and prescription can be discerned: the notion of measurement and measuring, of imposing a standard … This leads to the modern, nineteenth- and twentieth-century meaning of the term: the norm as signifying expected forms of social behaviour, based on sets of more or less implicit social rules that exist independently of individuals and exercise a coercive influence, with breaches of the norm being subject to sanctions.^23^

The emphasis on measurability resulted in the belief that true physical and moral health would be evident following the measurement of the body, revealing the importance that Western society placed on ‘normative appearance’.^24^ Although biology may have enacted some of the concerns related to the unhealthiness of excessive weight, it was the ‘verbal constructs, social contexts, and moral beliefs’ that connected weight with the ‘symbolic meanings associated with sets of symptoms and behaviours …’.^25^ As Annemarie Jutel suggests, ‘Deviance finds its place in disease’.^26^

Statistical ‘normality’ led to the utilisation of average measurements, which transcended into the commercial market through the standardisation of clothing. Standard sizing became a powerful normalising force in the mass consumer market, as it suggested that bodies should be changed to fit average clothing, rather than garments being made-to-measure for individual bodies.^27^ This standardisation furthered the belief in ‘normal’ and ‘abnormal’ shapes and sizes.^28^ However, this was not a new phenomenon. Women in the eighteenth and nineteenth centuries were expected to mould their bodies to conform to the figures and shapes that were deemed fashionable or beautiful at the time. They achieved this through the wearing of whalebone or steel corsets.^29^ Women manipulated their flesh to maintain a socially acceptable shape. For example, corsets enabled women to re-form their body shapes to adhere to a trend which lauded the combination of a narrow waist, with fuller hips and larger buttocks. Nevertheless, in the first half of the twentieth century, there was a shift towards it being the responsibility of the individual to cultivate their bodies within the socially accepted standards of the time. No longer was it acceptable to rely on material aids to achieve the desired body shape.

As the century progressed, instead of an unnatural, physically manipulated shape, there was promotion of a more natural body form. Elizabeth Ewing notes, ‘The waist was back in its normal place and, though there was no exaggeration of the figure at any point, curves were admitted to exist and allowed to be seen’.^30^ Furthermore, she suggests,

The fashions called for a long, slim look, and dieting became popular. Corsets, except for those who needed strict control, became progressively lighter, prettier and, as elastic materials improved, they were made more and more of elastic and without the menacing bones of past history … They did not exaggerate the bust, but improved its contours.^31^

Individuals were expected to maintain a physically and socially acceptable body through diet, nutrition and fitness activities. Therefore, the average body was measured through statistics, achieved through contemporary health or fitness advice, and signified social normality.

## The Promotion of Health and Beauty in British Culture

Nationwide anxieties associated with the degeneration of society were expressed through many facets of British culture, including the types of bodies that were displayed as part of the seaside freak show. The exhibition of people with abnormal weight highlighted that not achieving an average weight was physically dangerous and socially deviant.

Cultural engagement with contemporary health discourse was evident throughout all social classes and in a variety of entertainment spaces. British society was exposed to principles on health, body size, shape and beauty, from a variety of sources. These included magazines that provided health advice. The public was educated through these mediums and thereby, was made aware of changes to current trends and ideals regarding the body. Furthermore, middle- and working-class people absorbed health and beauty ideals through activities such as going to the cinema. Ross McKibbin argues that, ‘The cinema was the most important medium of popular culture in the period...’^32^ He exemplifies how cinema going influenced working-class women, who were fascinated by the glamour of Hollywood.^33^ McKibbin reveals,

For many women, almost independently of class, American movies were associated with ‘glamour’ … ‘Glamour’ implied a whole world – physical surroundings, clothes, cosmetics, luxury, - and ‘glamour’ was almost always the word used to describe it.^34^

Many women were influenced by the opulence of Hollywood actresses like Betty Grable, Doris Day, Rita Hayworth and Ava Gardner.^35^ In an increasingly consumer-focused society, women desired to emulate their luxurious lifestyles as far as financially possible. This meant shaping and dressing their bodies within the societal ideals that were visible to audiences at the cinema.^36^ Hollywood stars offered female spectators images of femininity that were unattainable for most. However, it provided them with a physical ideal that was healthy, feminine, glamorous and desirable. Popular actresses were the epitome of womanhood, enabling cinemagoers to identify with what they were seeing on screen. Jackie Stacey notes,

Not surprisingly, stars serve a normative function to the extent that they are often read as role models, contributing to the construction of the ideals of feminine attractiveness circulating in the culture at that time … Spectators often felt ‘unattractive’, ‘dowdy’, ‘plump’ and ‘gangly’ by comparison …^37^

Through identifying with ‘ideal’ actresses, the female population was able to assess their own bodies, health, and beauty. Consequently, women attempted to emulate what they saw at the cinema through engaging with the health or beauty discourse of the time. Through maintaining an acceptable body shape and styling it in a fashionable way, they could be more like the stars they saw in their favourite films.

One way of imitating what they saw on screen was to control their weight. Weight was central to British health and beauty discourse in the twentieth century. Zweiniger-Bargielowska states, ‘Doctors’ growing interest in obesity and the rise of a flourishing self-help reducing literature may seem incongruous for an era dominated by economic depression, unemployment, poverty and hunger marches’.^38^ However, while concern about weight-gain did not overshadow the Hungry England debate of the 1930s, it highlighted concerns surrounding unhealthy weights at both ends of the spectrum. The Inter-Departmental Committee on Physical Deterioration linked weight to health; it recognised weight as one of the methods through which health could be assessed. Concurrently, a plethora of contemporary dieting and nutritional publications showed weight to be central to healthiness.^39^ An article entitled ‘Weight and Health’ in the *Hartlepool Northern Daily Mail* on 5 May 1924 stated, ‘If a man’s weight is what it should be his health is all right’.^40^ Similarly, the *Yorkshire Evening Post* in 1926 noted, ‘There is no better way of gauging the progress of one’s health … than by regularly weighing oneself’.^41^ This demonstrated the view that sustaining an average weight was tantamount to overall physical health.

Displays of extreme weight in seaside freak shows capitalised on contemporary ideas surrounding weight and bodily size, to attract interest and debate at summer holiday resorts throughout the 1930s. They were seen as the antithesis of health and beauty. Audiences staring at those with bodies that lay outside average bodily standards were presented with the opportunity to understand their health, and the ways in which they needed to improve it. However, they were also able to assess their normality in relation to the unusual body on display. Therefore, displays of extreme weight conveyed messages to the audiences who viewed their exhibits. The public identified with those who presented themselves for entertainment, comparing their own bodies with those on display. They were able to place themselves on a scale of normality, and subsequently, engage with suitable activities to simultaneously improve their health and normalise their bodies.^42^

## Self-Starvation as a form of Entertainment

Staring at people with unusual bodies was a common form of entertainment at British seaside resorts. Those of extreme weights were particularly popular, with starving performers providing holidaymakers with a unique and titillating form of amusement. Walter Vandereycken and Ron van Deth understand self-starvation in terms of its social and cultural context. They suggest that the last 20 years of the nineteenth century proved most popular for starvation exhibits providing entertainment to audiences. Prior to fasting as an amusement, it was primarily seen as a divine sign from God and latterly, hunger striking was a method through which to take control in sociopolitical matters.^43^

Starvation as a form of entertainment was widespread in the late nineteenth and early twentieth centuries. Such exhibitions dominated coastal locations such as Blackpool, Margate and Southend-on-Sea, during the 1920s and 1930s, with numerous people displaying their fasting bodies for the amusement of onlookers. An article in *The Straits Times* provided details of these displays in Blackpool. It noted that,

Starving brides and bridegrooms, newly married couples, who it is admitted are mostly forced by poverty to make themselves a public show are enclosed in partitioned glass cases in three establishments on the Central Beach and are attempting to fast for periods of 30 and 32 days for wagers of £250. The show is the same at all three places. Each exposes the fasting pair to the gaze of the multitude. The couple lie in single beds, which are separated by a wall. The crowd flies round and looks through a glass top … Bride and bridegrooms straight from the altar starve for 30 days, says one poster. What courage! Will they do it … In front of the show are two waxwork ‘brides,’ one radiant, the other in draggled wedding garments and of corpse-line lineaments ‘after starving.’^44^

Holidaymakers were enticed to the exhibit by the melodramatic advertisements that updated them on the health of the participants. Messages chalked onto a blackboard declared, ‘How Long Can She Last’, ‘A Living Breathing Wonder’ and ‘The Pluckiest Girl in the World Now Lies Here In a Coffin’.^45^ This type of advertisement was reminiscent of posters from the Victorian period that used hyperbolic phrasing to attract audiences, denoting the continuation of freakery in seaside spaces.^46^

A newspaper article in the *West Lancashire Evening Gazette* published a photograph of a female ‘starving bride’ lying in a bed, appearing emaciated and weak, as onlookers discussed her body[Fig F0003].^47^ Undoubtedly one of the most controversial attractions, the ‘Starving Brides’ captivated holidaymakers.^48^ A sideshow worker revealed the popularity of the display.

‘You would be flabbergasted,’ an assistant at one show told me, ‘if you came here at the weekend. On Saturday and Sunday we have queues of people waiting to come in.’^49^

A contemporary equivalent to the itinerant freak shows of the Victorian period, spectators waited expectantly to view the unusual sight, and sought to better understand why the people inside the glass coffins might put their bodies at risk to perform for onlookers in this way. The exhibitions represented a plethora of concurrent societal debates, including the reasons why people were habitually fasting for the entertainment of others. *The Straits Times* reported that some of the participants were forced into displaying their bodies due to financial difficulties. The report suggested,

If anything is more nauseating than the shows themselves it is the cynical admissions that some of the men and women on view have been driven to make themselves public exhibits because of hard times, and the brazen cant that the showmen are really conferring a benefit on those unfortunate souls by giving them the chance of gaining money in this way.^50^

The exhibits produced polemic disputes about the morality of displaying vulnerable individuals as part of the freak show. The showmen responsible for the inauguration of the exhibitions supported their decision to provide performers with employment.

‘They would starve on the streets, anyhow,’ one proprietor told me. ‘Surely it is better for them to starve in a glass cabinet and get an opportunity to set themselves up in life. I give generous compensation even if they cannot last out the full time.’^51^

The showmen endorsed their decision to display the fasting artists, as it afforded performers the prospect of providing for their fiscal needs. The couples had no other option, if they wished to marry and therefore, the proprietors validated their own morality by arguing that they were giving them the opportunity to make a substantial amount of money. However, controversy was unrelenting, as some regarded the displays as evidence of the privileged exploiting the weakest and most vulnerable in society. Moreover, they put the entertainers’ lives in danger and threatened their individual health. The display revealed the financial desperation that some people experienced and the lengths they would go to, in order to earn money. Therefore, it exposed fears surrounding the economic health of the nation, and subsequently, the physical deterioration of the British race in the first half of the twentieth century.

**Figure 3. F0003:**
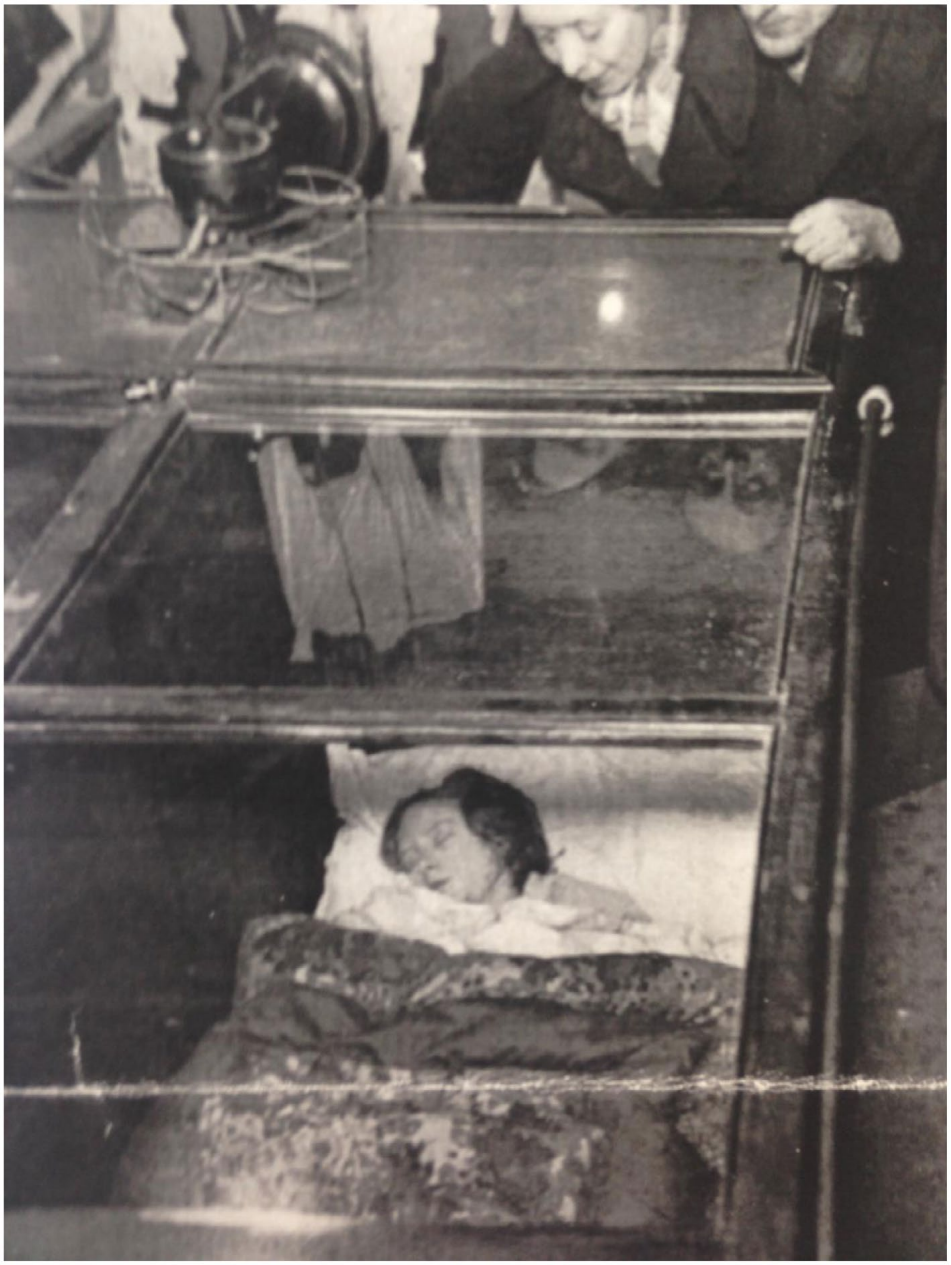
Photograph of the ‘Starving Brides’, Blackpool, The Local and Family History Centre, Blackpool Central Library, Cyril Critchlow Collection, The Starving Brides file.

Copyright: Getty Images.

Although a macabre form of entertainment, displays of emaciated individuals also had an educational function and warned the public of the dangers of malnutrition. The *Biggleswade Chronicle* reported that outside the exhibit,

were life-size cut-out pictures of a blushing bride (‘as she was’) followed by others in gradual stages of deterioration until a final cadaverous representation was described ‘as she is now.’ On the ground floor a long queue was lined up to pay 2d. to look at this remarkable spectacle. Apparently there was a wager or something of that sort, for posted up on a blackboard near the entrance was a notice to the effect that ‘as the starving bride cannot now win the £250, to-day is positively the last day of exhibition.’ And that ‘all profits of this day will be given to the starving brides.’ Poor Girl!^52^

As well as life-size cut-outs of the starvers, morbidly curious observers were confronted with waxworks depicting emaciated brides suffering from the effects of starvation.

The model representing the bride was purchased from a local Ladies Outfitter and has the following notice attached, ‘As this bonny girl thinks she will appear on her wedding day.’ The Head of the ‘Emaciated corpse like figure’ was made by a local amateur wax molder and bears the following placard ‘But we know she will look like this’.^53^

This suggests that some of the brides were starving themselves prior to their marriage, perhaps in a bid to lose weight for their wedding day, whilst others were starving themselves for money following their nuptials.

The life-size cut-outs and daily updates on the health of the acts showed the public the harmful effect of malnutrition on the body. The pictorial representations presented a visual warning of the results of poor nutritional intake; they were documentation of the stages of deterioration that people went through as a result of starvation. The public was responsible for maintaining their health and the exhibition cautioned them that malnourishment led to death. The exhibitions subtly encouraged people to take control of their health. Through assessing the cut-outs and the displays, audiences were able to measure themselves against the ‘ill-health’ or ‘abnormality’ of the emaciated body. Ultimately, the public could either reassure themselves of their personal health or assess their need to improve their nutritional intake; otherwise, much like the acts they were viewing, they would risk death.

Death was an intrinsic aspect of the construction of ‘starving bride’ exhibitions. The physical display cabinet was described as a ‘coffin’, demonstrating the morbidity accompanying the display.

The size of the coffin is approximately 8ft. long, by 3 ft. wide, and 3ft. 6 ins. in depth, and has a glass top. Inside, at the head, there is a chemical incinerator, the top of which forms a shelf and on this stands bottles of lemonade and packets of cigarettes.^54^

The depiction of the starving brides in a coffin, a poignant and universal symbol of death, revealed that starvation and death were interrelated. The displays capitalised upon the cultural fascination and curiosity surrounding death. However, they also served as a definitive warning of the seriousness of malnutrition, clearly linking starvation and mortality to holidaymakers.

Death was not just symbolic in starvation exhibitions. When champion faster, Ricardo Sacco, died from stomach problems following a 40-day fast, it demonstrated how precarious such displays were to the health of participants. The morality of self-starvation exhibits was questioned. Sacco fasted in numerous seaside locations, including Blackpool and Southend, in the early twentieth century, losing approximately 2 stone throughout the duration of his fasts.^55^ However, in 1929, Sacco died following 65 days of self-starvation. An article in the *Dundee Courier* reported,

His friends say that his death was not caused by fasting, but by an internal complaint. During his last fast at Blackpool he had stated that he would not enter upon another similar feat, but intended to retire … He was extremely emaciated and had been confined to his bed for several weeks. In his youth he had been a fine specimen of manhood, but his many prolonged fasts during which he lived in glass cases, had reduced him to little more than a skeleton … When fasting he took only soda water and frequently smoked cigarettes.^56^

The report suggested that, in spite of the longevity of his starvation and his stomach complaints, his family and friends wished to avoid the association between his career as a champion faster and his untimely death.^57^ Notwithstanding their denial, it seems apparent that there was a link between the weakening of his body through malnutrition and the ‘internal complaint’ that led to his death.

There were serious concerns over the health of participants in starvation displays and newspapers debated the morality of such exhibitions. The *Daily Mail* described the ‘starving brides’ as ‘perfectly horrid, un-Christian and beastly shows’.^58^ Additionally, religious leaders petitioned the Home Office to prevent people starving themselves as entertainment. One letter from a Church of England Vicar stated,

… that such an abuse of the Body, is little short of deliberate suicide … Apart from that we feel that the Human Body is sacred, and should not be so abused. I write very humbly to ask, IF YOU CAN GIVE ME DETAILS OF ANY LAW BY WHICH WE CAN PROCEED TO STOP THE NAUSEATING SPECTACLE.^59^

This portrayed the varied and widespread concern surrounding starvation and malnutrition. During the 1930s, there was significant debate centred on the undernourishment of the working classes, whom, despite working long hours, could not afford to eat enough food of sufficient nutritional value. The Greenwood Committee was established to examine malnutrition in the working classes, attesting to the concern surrounding the health and well-being of the British public. However, minutes from the committee meetings expose the distinct lack of clarity related to the physical evidence or symptoms of malnutrition. Problematically, doctors could not clearly define ‘malnutrition’ and therefore, the reports and surveys carried out by the state were limited in their effectiveness to give an overall picture of the health of the population.

A vague description of malnourishment was reported in the committee minutes. It declared that, ‘Malnutrition is not an entity but is an agglomeration of metabolic disturbances due to improper and defective dietaries and consequently its characteristics may be expected to vary in accordance with the nature of dietary defect’.^60^ However, it was agreed amongst the members that some diseases were the direct result of malnourishment, such as rickets and scurvy.

The investigations carried out by the committee found that children were not extraordinarily affected by malnutrition, apart from a number of exceptions. Nevertheless, it was unclear to what extent malnutrition affected the public because of the imprecise nature of what constituted malnutrition in the first place. The committee proposed that education was an ‘effective weapon’ to attack the problem of malnourishment and the diseases caused by insufficient nutrition. They suggested that doctors, teachers, ‘other public-spirited persons’ should be responsible for the dissemination of information regarding health, diet and proper nourishment.^61^ This revealed the complexities and difficulties associated with improving the health and diet of the public. Fundamentally, it demonstrated a lack of engagement from healthcare professionals with improving diet.

There was extensive contemporary debate about the extent of malnutrition throughout Britain in the 1930s. Yet, Charles Webster argues that ‘The statistics again point to an improvement in the national health and physical well-being of the population. Death rates declined, children were on average fatter and healthier than their parents had been, and the worst forms of malnutritional diseases, such as rickets and scurvy, had all but disappeared by the second world war’.^62^ However, Juliet Gardner reveals that women often died as a result of giving up food for their family and sacrificed their lives for the sake of their children, suggesting a more widespread problem.^63^ The press published stories throughout the thirties and the Week-End Review launched a ‘Hungry England’ Inquiry in spring 1933. ‘They found that unemployment relief payments were insufficient to provide the minimum diet for a family recommended by the recently established Advisory Committee on Nutrition set up by the Ministry of Health…’^64^ Therefore, it is clear that in some sections of British society there was evidence of malnourishment but the degree of this was ambiguous.

The anxieties about malnutrition were evident in the debates about starvation performers and the local authorities called into question the appropriateness of such displays. A member of the Blackpool Town Council stated,

My view is that when people come to Blackpool they come for a healthy and invigorating holiday, and to see fat women on show and thin ones who are starving in coffins and barrels, must be, to say the least of it, not nice.^65^

There were a myriad of reasons that people regarded starvation displays as objectionable. Not only did actively starving oneself go against contemporary health advice but it also encouraged the perception of Blackpool as the centre of objectionable entertainments. Exhibitors endangered their health for money, which raised apprehensions about the economic circumstances in Britain; starvers had to risk death in order to provide for their physiological needs. However, through starving, they were not able to maintain optimum physical health. Therefore, broader societal concerns were revealed through starvation exhibits. Namely, the public had to resort to drastic measures, including starvation, to meet the needs of their families, but even this was not enough to preserve the health of the nation.

## Excess Weight as a Form of Entertainment

Whilst unemployed members of British society, of which there were 3 million in 1931, experienced malnutrition, obesity was more prevalent in the middle classes.^66^ Although starvation displays reflected the understanding that undernourishment was precarious, audiences were able to comprehend the risks of overindulgence through viewing those with abnormally large bodies. Zweiniger-Bargielowska points out,

The concurrence of obesity with extensive under-nutrition exemplifies the social and economic inequality of interwar Britain, characterized by the emergence of affluence within the middle classes and persistent poverty among sections of the working class. The disparate food systems of the middle and working classes represented opposing poles and yet, dietary patterns and habits of both groups were also perceived to exhibit common elements because fat, emaciated and stunted bodies deviated from conceptions of the normal.^67^

Fat men and women represented the diverse food patterns of the middle and working classes, and were presented as the antithesis of starvation exhibitions. This revealed to holidaymakers that neither polarities were acceptable and while they should not overly restrict their food intake, neither should they overindulge.

In 1937, E. E. Claxton revealed, in his book *Weight Reduction: Diet and Dishes*, that health professionals looked to the freak show to educate the public in leading a healthy lifestyle. He exemplified Daniel Lambert, one of the most famous fat men in 1,770, as an example of being grossly overweight.^68^ Claxton noted,

Let us first take some of the minor disadvantages, and with them we may remember that fatness has been a butt for humour from time immemorial. Why do we laugh or want to laugh at the fat man? The fat man is the one exception that we allow ourselves for laughter directed against or caused by deformity. A hundred or more years ago almost any physical abnormality was an object of humour. The dwarf, the giant, the hunchback, the cripple, the thin man, the fat man, and so on, were all considered a great joke. These curiosities and anomalies survive still for the amusement of morbid onlookers at side shows in fairs and circuses, but laughter at the expense of the unfortunate victims is now confined only to the obese, and even that is considered in these days as questionable taste… we must still admit, however, that a fat man or woman looks funny.^69^

This indicated that fat people remained the object of humour in the late 1930s, demonstrating the social repercussions for those who did not keep their bodies within the confines of normality.

Displays of people such as Lenny Mason, the ‘Leicester Fat Boy’ and Miss Rosie, a popular fat lady, alongside those participating in long fasts, warned the public to keep their bodies within the average statistical sizes. They should be neither too thin, nor too fat. It was well known that starvation risked death but obesity had physical and social consequences too.

Lenny Mason, the Leicester Fat Boy, exhibited his obese body on Blackpool’s Central Beach.^70^ He was reported to be the ‘WORLD’S FATTEST BOY’.^71^ An article entitled ‘What will the food controller say?’ offered a £100 reward for anyone who could find a rival to him.^72^ This revealed that Mason was particularly large for his age and that it was seemingly impossible to find anyone who could challenge his size. Advertisements that promoted his display were particularly focused on the measurements of his body. One newspaper reported,

Lenny Mason, of Leicester, is said to be the fattest boy in the world. He is 16 years of age, and 5 feet 3 inches in height but his waist measurement is 64 ½ inches and his arm measures 23 inches round. He weighed 27 stone when he was fourteen years of age and his present weight is thirty stone.^73^

The interest in the circumference of his waist and arms echoed the greater focus on measurements as an indicator of health. Dieting became increasingly popular in the second half of the nineteenth century and measurements became a method through which to assess the success of the dieter. Subsequently, the public became more aware that health and a long life were the benefits of maintaining good physical shape. People were educated that health was based on a number of factors, including eating well and exercising sufficiently.

Greater scientific understanding of the way the body worked was followed by contemporary dietary advice. ‘Reducing manuals… recommended a modified diet, physical culture, moderation with regard to alcohol and tobacco consumption, thorough mastication, and regular evacuation of the bowels’.^74^ Additionally, local and national newspapers contained advice that suggested ways that the public could combat obesity and ill health. One such example recommended,

If you suffer from obesity, you need not be afraid of eating plenty of butter … This does not induce a tendency to obesity as so many people suppose. Your diet should be a well-selected one. Eat toast instead of bread; eat plenty of green vegetables and fruit. Avoid too many soups. Take meat in moderation, and as much fish and poultry as you like, with the exception of shell-fish and salmon. Take as little sugar in your food and beverages as possible. Another effective plan in keeping away obesity is to never drink with your meals. Take a glass of warm water half an hour before meals and a glass of cold water half an hour afterwards.^75^

Health advice followed the latest medical discoveries and followed well-being trends. As the twentieth century continued, people were increasingly aware of the relationship between health, nutrition and exercise. Newspapers described ways that people could improve their health through physical exertion.

Walking is the best example in the treatment of obesity and should be taken after food rather than before, because otherwise it may lead to an increased appetite, and the patient may be inclined to eat more than he otherwise would. In warm weather swimming is a most amicable form of exercise, combining both the exercise of the muscles and the advantage of bathing. The patient should be regularly weighed and measured, so as to determine the results which are being obtained. A diminution in weight should not be effected too rapidly.^76^

This exposed a continued fascination with statistical measurements, body size and weight. The public was provided with the information to aid their reducing endeavours and improve their physical health.

As well as the physical implications of obesity, there were also social repercussions to being overweight. Sander L. Gilman contends, ‘By the end of the eighteenth century, a new idea enters the understanding of the cause and the treatment of the obese, the failure of mind rather than the failure of the body’.^77^ Fatness was regarded as resulting from a breakdown of morality. Therefore, there was stigma attached to men and women who struggled with weight-related issues.^78^ Although the social construction of corpulent individuals differed depending on gender, at its core was the belief that extreme fatness signified socially problematic individuals. This suggested that people with unusually large bodies were perceived as physically and socially inferior to those who were able to control their body weight. The body was symbiotic with the construction of identity; freakery displays encouraged spectators to remain within normative social boundaries through ‘othering’ unusual bodies and constructing them as ‘abnormal’. The social construction of freak show acts targeted men and women in different ways to encourage them to reside within heteronormative gender roles.

The display of fat boys such as Lenny Mason can be analysed, ‘against the backdrop of dominant codes of manliness and a wider debate about the physical fitness of the British race’ to understand how audiences perceived their displays.^79^ A *carte de visit* of Mason’s exhibit showed that his arms and legs were uncovered in order to accentuate his corpulence. Moreover, he was portrayed wearing a tunic – a particularly feminine piece of clothing. The skin of fat people was often revealed so that audiences could stare at the extent of their fatness and measure themselves in relation to the obese body on show.^80^

Nevertheless, the display of Mason’s skin and his presentation in feminine clothing provided humour for the audiences viewing his exhibit. Corpulent men contrasted with the Greek ideals of beauty that stressed muscular strength, athleticism, and pride in appearance. Bodily weakness was observed as effeminate and male fatness was perceived as humorous. In 1926, Leonard Williams stated,

The unlovely condition called corpulence, or obesity, has been divided into three stages, known respectively as the enviable, the comical, and the pitiable. Such classification is based upon a false estimate of values; for no case of obesity is enviable, most of them are in a sense comical, and all are pitiable.^81^

This demonstrated that fat people were regarded in two main ways; as humorous or pathetic, both of which defied conceptualisations of masculinity between 1920 and 1940. Zweiniger-Bargielowska points out that obesity conflicted with ‘hegemonic masculinity’ and men were ridiculed for it.^82^ She reveals that ‘The association of obesity and fat with humiliation, ridicule, or pity was perhaps an even greater incentive to reduce’.^83^

By linking fatness so closely with identity and humour, it associated spectacle with shame. Lenny Mason demonstrated what men should not be; unfit, uncontrolled and gluttonous. Rather they were to partake in exercise, eat well, and maintain their strength. Mason’s body stimulated audiences to procure their physical strength, through inducing fear and relieving it. Mason’s fatness encouraged people to normalise themselves in relation to him through better body management. Through staring at the unusual body and understanding its physical and social ‘abnormality’, audiences could then assess their own bodies and ‘normality’ by comparing it to the person on display. People became afraid of the consequences of overindulging, and controlled their food intake in order to remain within average statistical norms.

Men were expected to maintain their manliness through physical strength and fitness, and the ‘cultivation of a fit male body’ was central to British citizenship and a response to ‘the needs of the British Empire’.^84^ A contemporary book on obesity, published in 1926 noted, ‘Now homosexuals of both types, male and female, show a definite tendency to obesity’.^85^ This shows how the fears surrounding obesity and the feminisation of the male body reveal a greater moral struggle. To society, male obesity signified an inability to control sexual desire, and the fat body was associated with homosexuality. However, while some people partook in activities that facilitated a reduction of their weight, to improve their physical appearance, others embraced their ‘abnormality’ and displayed themselves in front of paying audiences. They asserted agency over their physically different bodies by exhibiting them to the public.

Similarly, displays of fat ladies encouraged female audience members to reside within social codes of normality. Like Lenny Mason, Miss Rosie was displayed in Blackpool in 1930, weighing almost 36 stone. The ‘othering’ of Miss Rosie’s body as abnormally large, demonstrated that women should compare their bodies to her body, adjusting their weight accordingly. However, her exhibit also promoted domesticity and appropriate sexual behaviour. As seen through Miss Rosie’s *carte de visit*, the fat lady was simultaneously feminised and sexualised, and portrayed a number of gendered ideals to female holidaymakers.^86^ Miss Rosie was depicted as overtly feminine; she held flowers in her hand, which represented her femininity.^87^ Additionally, a newspaper report on Miss Rosie’s exhibit stated, ‘She is engaged at needlework, but at your entrance a smile creases her face, and almost hides her eyes in rolls of fat’.^88^ Miss Rosie was both costumed in feminine clothing and displayed occupied with pastimes that were typically associated with women, which revealed to the audience that female holidaymakers were expected to reflect upon her exhibition. It showed that despite her physically deviant body, she still attempted to participate in socially acceptable behaviours.

While Miss Rosie reinforced gender stereotypes, she simultaneously defied them. A newspaper report that detailed her exhibition disclosed, ‘She wears a flamboyant evening gown, sleeveless and cut perilously low…’ which revealed the inherent concern with the sexual deviance of the fat lady.^89^ The uncovering of skin was considered inappropriate, even within the relaxed boundaries of British seaside resorts. The sexualisation of Miss Rosie, through her revealing dress and the unveiling of her excess skin, demonstrated to female spectators that physical deviance could lead to social deviance. Therefore, female audience members needed to control their bodies and desires. Female freakery exhibits sought to encourage women who attended the display to keep their bodies within the confines of physical and sexual ‘normality’, in the hope that this would encourage an improvement in the morality of women. This echoed contemporary fears surrounding the moral degeneration of the British nation.

The displays of fat people, much like the exhibitions of extraordinarily thin people, revealed the concerns surrounding the physical and moral health of the nation. Freak shows were constructed utilising public discourse surrounding weight, healthiness, hunger, and obesity. Those who were not keeping their bodies within average statistical measurements were regarded as deviant, as they defied what were socially and medically accepted conceptions of ‘normality’. They were represented as morally and socially inferior to people whose bodies adhered to the ‘normal’ or ‘average’ cultural stereotypes. Therefore, it is evident that freak shows, through ‘othering’ those with unusual physiques, showed audiences the consequences of not maintaining a statistically average body and subsequently promoted health, body management and social normality.

## Conclusion

Freak shows remained a popular part of British culture well into the twentieth century. Although they became less prevalent within modern, metropolitan spaces, they continued to be a central part of culture at the margins of society. Seaside resorts provided a space in which displays of freakery thrived and continued to be an enjoyable entertainment for holidaymakers. Removed from the social constraints of everyday life, people were permitted to stare at those who were different as part of their leisure pursuits. However, like freak shows in the nineteenth century, twentieth century displays of unusual bodies reflected many complex public discourses. As demonstrated through the case studies of starvation and obesity, bodily displays continued to reflect health and fitness culture, including constructions of the ‘normal’ and ‘abnormal’ body.

Popular health discourse was directly related to concerns about the state of ‘Hungry England’, as well as the rising obesity levels of the middle classes. This was evidence of the growing inequalities between the upper middle classes and the working classes. However, it also demonstrated the issues with not obtaining optimum health. The public was encouraged to take control of their health, and this included maintaining an average, acceptable, and healthy weight. Health and beauty discourse was evident in various areas of public life, including popular dieting literature, newspapers and in entertainment.

The ‘Starving Brides’ proved to be a controversial display of freakery. They were constructed with concepts of malnutrition and intensified fears surrounding the physical deterioration of the British race. Whilst the display warned people of the consequences of inadequate nutrition, the starving brides also exposed the lengths to which some people went to provide for their families. At the opposite end of the spectrum, Lenny Mason portrayed to male audiences, in particular, that they should avoid overindulgence and maintain a strong, healthy physique. Miss Rosie revealed to female spectators that they should not only retain a healthy body but should additionally remain within heteronormative social boundaries. As such, holidaymakers were able to place themselves on a scale of normality and therein better understand their own bodies, health and physical fitness. Although some became increasingly disconcerted with the display of unusual bodies, freak shows continued to provide entertainment, education, and spectacle in seaside resorts until the late 1940s when the rise of holidaying abroad affected the attractions available to holidaymakers in Britain, which consequently impacted on the display of unusual bodies.

## Disclosure statement

No potential conflict of interest was reported by the author.

## Notes on contributor

***Emma Purce*** is a Wellcome Trust PhD Student at the School of History at the University of Kent.

## Funding

This work was supported by the Wellcome Trust [Grant number 104892/Z/14/Z].

## Notes

1.
*Lancashire Evening Post*, Saturday 23 September 1933, p. 6.

2.
*Biggleswade Chronicle*, Friday 25 August 1933, p. 3.

3. Figure 1, *Carte de Visit* for Lenny Mason, Leicester Fat Boy, Blackpool, The Local and Family History Centre, Blackpool Central Library, Cyril Critchlow Collection, Vol. 163; Figure 2, *Carte de Visit* for Miss Rosie, The Fat Lady, Wellcome Library London, EPH WD. Freaks:1:1 (Nottingham, c.1900s).

4. Nadja Durbach, *Spectacle of Deformity: Freak Shows and Modern British Culture* (London, 2010), p. 14.

5. Nadja Durbach, *Spectacle of Deformity*, p. 1. See also Robert Bogdan, *Freak Show: Presenting Human Oddities for Amusement and Profit* (London, 1990) and Rosemarie Garland Thomson, *Freakery: Cultural Spectacles of the Extraordinary Body* (New York, 1996).

6. Durbach, *Spectacle of Deformity,* p. 1.

7. Ibid., p. xi.

8. Nadja Durbach, *Spectacle of Deformity: Freak Shows, and Modern British Culture* (London, 2010), p. 171.

9. John K. Walton, *The British Seaside Holiday: Holidays and Resorts in the Twentieth Century* (Manchester, 2000), p. 3.

10. Josephine Kane, *The Architecture of Pleasure: British Amusement Parks 1900*–*1939* (Surrey, 2013) pp. 97–98.

11. John K. Walton, *Blackpool* (Edinburgh, 1998), p. 1.

12. Ibid., p. 5.

13. Joanna Bourke, *Dismembering the Male: Men’s Bodies, Britain and the Great War* (London, 1996), p. 172.

14. Roderick Floud, Kenneth Wachter, and Annabel Gregory, *Height, Health and History: Nutritional Status in the United Kingdom, 1,750*–*1980* (Cambridge, 1990), p. 63.

15. Anne Hardy, *Health and Medicine in Britain since 1860* (Basingstoke, 2000), p. 77.

16. Vicky Long and Hilary Marland, ‘From Danger and Motherhood to Health and Beauty: Health Advice for the Factory Girl in Early Twentieth-Century Britain’, *Twentieth Century British History*, 20, No. 4 (2009), p. 464–465.

17. Anne Hardy, *Health and Medicine in Britain since 1860* (Basingstoke, 2000), p. 77.

18. This became even more prevalent following the Holidays with Pay Act in 1938. More people were freed from work and able to experience holidays by the coast.

19. Many of these ideas were popularized by the Sunlight League (established in 1924) and *Man and Sunlight* (published in 1927).

20. John K. Walton, *Fish and Chips and the British Working Class, 1870*–*1940* (Leicester, 2000), p. 13.

21. The development Adolphe Quetelet’s ‘average man’ in 1835, generated a methodological framework through which ‘normal’ physicality could be distinguished, including what constituted an average body weight. Additionally, Quetelet’s statistical methods were used to define normal physical and mental features, which were subsequently implemented to distinguish ‘normal’ and ‘abnormal’ social behaviour. Accordingly, his ideas promoted the concept that those with ‘abnormal’ bodies were socially transgressive, and thus, subject to ‘enfreakment’.

22. Waltraud Ernst, ‘The Normal and the Abnormal: Reflections of Norms and Normativity’, in Waltraud Ernst (ed.), *Histories of the Normal and the Abnormal: Social and cultural histories of norms and normativity* (Oxon, 2006), p. 2.

23. Ernst, ‘The Normal and the Abnormal: Reflections of Norms and Normativity’, p. 2.

24. Annemarie Jutel ‘The Emergence of Overweight as a Disease Entity: Measuring up Normality’, *Social Science and Medicine*, 63 (2006), p. 2269.

25. Ibid.

26. Ibid.

27. Alison Matthews David, ‘Made to Measure? Tailoring and the ‘Normal’ Body in Nineteenth-Century France’, in Waltraud Ernst (ed.), *Histories of the Normal and the Abnormal: Social and Cultural Histories of Norms and Normativity* (Oxon, 2006), p. 159.

28. Elizabeth Ewing, *History of Twentieth Century Fashion* (London, 1986), p. 119, 127.

29. Charlotte Delory as quoted in Denis Bruna (ed.), *Fashioning the Body: An Intimate History of the Silhouette* (London, 2015), p. 229.

30. Ewing, *History of Twentieth Century Fashion*, p. 112; Changes to fashion (including underwear fashion) reflected a number of social changes. For example, clothing changed to facilitate women in the workplace, female sporting activities and the more acceptable exposure of skin to sunlight.

31. Ibid., pp. 108–109.

32. Ross McKibbin, *Classes and Cultures: England 1918*–*1951*, p. 419.

33. Ibid., p. 421.

34. Ibid., p.432.

35. See Melanie Williams and Ellen Wright, ‘Betty Grable: An American Icon in Wartime Britain’, *Historical Journal of Film, Radio, and Television*, 31, No. 4 (2011), pp. 543–559; Working class women would have been further influenced by Hollywood and Movie Magazines, such as *Picturegoer* or *Film Weekly*.

36. Body shape went under its own transformations throughout the twentieth century, with different shapes and sizes popular at different times.

37. Jackie Stacey, *Star Gazing: Hollywood cinema and the female spectatorship* (New York, 1994), p. 152.

38. Ina Zweiniger-Bargielowska, ‘Slimming Through the Depression: Obesity and Reducing Culture in Interwar Britain’, p. 177.

39. Examples of health publications that mention weight: E. E. Claxton, *Weight Reduction: Diet and Dishes* (London, 1937); Yorke Davies, *Foods for the Fat: The Dietetic Cure of Obesity* (London, 1904); Ettie Hornibrook, *Stand Up and Slim Down* (London, 1934).

40. ‘Weight and Health’, *Hartlepool Northern Daily Mail*, 5th May 1924, p. 4.

41. ‘Weight and Health’, *Yorkshire Evening Post*, 1926, p. 8.

42. However, food rationing in the Second World War had a significant impact on dieting culture due to the introduction of rationing and nationwide austerity in Britain.

43. For example, self-starvation was used by Suffragettes in order to secure votes for women.

44. ‘Brides Starve in Coffins’, *The Straits Times*, 28 October 1934, p. 7; *Lancashire Evening Post*, Saturday 08 September 1934, p. 5.

45. The National Archives Entertainment: Exhibitions of persons fasting (Ref: HO 45/16275). Kew, London.

46. Notably, there was less promotion of starving husbands, although they also fasted for the amusement of others. Male starvation as entertainment was less common than female starvation for a number of reasons. Firstly, thin bodies were more acceptable for women than for men. Rather, male bodies were supposed to be muscular, fit, and strong as the strength of the male body was a reflection of the strength of the nation. Muscular Christianity advocated piety, physical health, and wellbeing. Male starvers directly contradicted these beliefs. Perhaps, therefore, it was regarded as less appropriate for men to perform this type of entertainment.

47. Figure 3, The Local and Family History Centre, Blackpool Central Library, Cyril Critchlow Collection. Newspaper Clippings. *‘Gazette and Herald 09/08/1930’.*

48. Although self-starvation is currently understood as a psychological condition (Anorexia Nervosa), it must be understood in terms of motivations within its social, cultural, and economic context.

49. ‘Brides Starve in Coffins’, *The Straits Times*, 28 October 1934, p. 7.

50. Ibid.

51. Ibid.

52.
*Biggleswade Chronicle*, Friday 25 August 1933, p. 3.

53. The National Archives, Entertainment: Exhibitions of persons fasting (Ref: HO 45/16275). Kew, London.

54. Ibid.

55.
*Motherwell Times*, Friday 09 September 1927, p. 6.

56.
*Dundee Courier*, Tuesday 05 November 1929, p. 9.

57. This can be attributed to the association between starvation and suicide. Suicide was illegal in Britain until the Suicide Act of 1961.

58.
*Hull Daily Mail*, Saturday 12 October 1934, p. 7.

59. The National Archives, Entertainment: Exhibitions of persons fasting (Ref: HO 45/16275).

60. The National Archives, Diet and Nutrition Advisory Committee (Greenwood Committee) Standards of Malnutrition. (Ref: MH 56/53).

61. Ibid.

62. Charles Webster, ‘Hungry or Healthy Thirties?’, *History Workshop Journal*, 13, No. 1 (1982), p. 110–111.

63. Juliet Gardner, *The Thirties: An Intimate History* (London, 2011), p. 67.

64. Ibid.

65.
*Lancashire Evening Post*, Saturday 08 September 1934, p. 5.

66. Ina Zweiniger-Bargielowska, ‘The Culture of the Abdomen: Obesity and Reducing in Britain, circa 1900–1939’, *Journal of British Studies*. 44, No. 2 (2005), p. 242; The upper classes were not as affected by weight related issues because, although they had an abundance of food, they simultaneously engaged in rigorous physical activities that balanced out their consumption, meaning that they maintained an average body weight; Anne Hardy, *Health and Medicine in Britain since 1860* (Basingstoke, 2000), p. 78.

67. Ina Zweiniger-Bargielowska, ‘Slimming Through the Depression: Obesity and Reducing Culture in Interwar Britain’, p. 188.

68. E. E. Claxton, *Weight Reduction: Diet and Dishes* (London, 1937), p. 2.

69. Ibid., p. 14.

70. Figure 1, *Carte de Visit* for Lenny Mason, Leicester Fat Boy, Blackpool, The Local and Family History Centre, Blackpool Central Library, Cyril Critchlow Collection, Vol. 163.

71.
*Dundee Evening Telegraph*, Thursday 30 October 1919, p. 8.

72.
*Derby Daily Telegraph*, Friday 22 February 1918, p. 1.

73.
*Dundee Evening Telegraph*, Thursday 30 October 1919, p. 8.

74. Ina Zweiniger-Bargielowska, ‘The Culture of the Abdomen: Obesity and Reducing in Britain, circa 1900–1939’, *Journal of British Studies*, 44, No. 2 (April, 2005), p. 267.

75.
*Framlingham Weekly News*, Saturday 21 July 1917, p. 2.

76.
*Rochdale Observer*, Saturday 28 August 1915, p. 2.

77. Sander L. Gilman, *Obesity: The Biography* (Oxford, 2010), p. 59.

78. Ibid., p. 63.

79. See Joanna Bourke, *Dismembering the Male: Men’s Bodies, Britain and the Great War* (London, 1999).

80. Firstly, prior to the twentieth century, obesity signified strength and manliness, so displays of fat boys reflected the changing construction of the ‘ideal’ body. Obesity was no longer considered a sign of strength, but feminine and thus, weak. Secondly, twentieth century audiences would not have seen obesity on such a scale as this in everyday life.

81. Leonard Williams, *Obesity* (1926), p. 1.

82. Ina Zweiniger-Bargielowska, ‘The Culture of the Abdomen: Obesity and Reducing in Britain, circa 1900–1939’, *Journal of British Studies*, 44, No. 2 (2005), p. 262.

83. Ibid., p. 264.

84. Ina Zweinger-Bargielowska, ‘Building a British Superman: Physical Culture in Interwar Britain’, p. 596.

85. Williams, *Obesity* (1926), p. 72.

86. For male holidaymakers, they may have regarded her as attractive or sexual. Female holidaymakers were more likely to compare their body to her body, assessing their ‘normality’ or ‘abnormality’.

87. Figure 2, *Carte de Visit* for Miss Rosie, c. 1900, Wellcome Library, London. EPH WD. Freaks:1:1.

88. Figure 2, *Carte de Visit* for Miss Rosie, c. 1900, Wellcome Library, London. EPH WD. Freaks:1:1.

89. The Local and Family History Centre, Blackpool Central Library, Cyril Critchlow Collection. Newspaper Clippings. *‘Gazette and Herald 09/08/1930’.*

